# Identification of the novel prognostic biomarker, MLLT11, reveals its relationship with immune checkpoint markers in glioma

**DOI:** 10.3389/fonc.2022.889351

**Published:** 2022-08-12

**Authors:** Long Chen, Zujian Xiong, Hongyu Zhao, Chubei Teng, Hongwei Liu, Qi Huang, Siyi Wanggou, Xuejun Li

**Affiliations:** ^1^ Department of Neurosurgery, Xiangya Hospital, Central South University, Changsha, China; ^2^ Xiangya School of Medicine, Central South University, Changsha, China; ^3^ Hunan International Scientific and Technological Cooperation Base of Brain Tumor Research, Xiangya Hospital, Central South University, Changsha, China

**Keywords:** glioma, bioinformatics, single-cell RNA sequencing, tumoral heterogeneity, immune infiltration

## Abstract

**Aim:**

This study aimed to explore the expression pattern of *MLLT11* under different pathological features, evaluate its prognostic value for glioma patients, reveal the relationship between *MLLT11* mRNA expression and immune cell infiltration in the tumor microenvironment (TME), and provide more evidence for the molecular diagnosis of glioma and immunotherapy.

**Methods:**

Using large-scale bioinformatic approach and RNA sequencing (RNA-seq) data from public databases The Cancer Genome Atlas (TCGA), Chinese Glioma Genome Atlas (CGGA), and The Gene Expression Omnibus (GEO)), we investigated the relationship between *MLLT11* mRNA levels and pathologic characteristics. The distribution in the different subtypes was observed based on Verhaak bulk and Neftel single-cell classification. Then, Gene Ontology (GO) and Kyoto Encyclopedia of Genes and Genomes (KEGG) enrichment analysis were used for bioinformatic analysis. Kaplan–Meier survival analysis and Cox regression analysis were used for survival analysis. Correlation analyses were performed between *MLLT11* expression and 22 immune cells and immune checkpoints in the TME.

**Results:**

We found that *MLLT11* expression is decreased in high-grade glioma tissues; we further verified this result by RT­PCR, Western blotting, and immunohistochemistry using our clinical samples. According to the Verhaak classification, high *MLLT11* expression is mostly clustered in pro-neutral (PN) and neutral (NE) subtypes, while in the Neftel classification, *MLLT11* mainly clustered in neural progenitor-like (NPC-like) neoplastic cells. Survival analysis revealed that low levels of *MLLT11* expression are associated with a poorer prognosis; *MLLT11* was identified as an independent prognostic factor in multivariate Cox regression analyses. Functional enrichment analyses of *MLLT11* with correlated expression indicated that low *MLLT11* expression is associated with the biological process related to the extracellular matrix, and the high expression group is related to the synaptic structure. Correlation analyses suggest that declined *MLLT11* expression is associated with increased macrophage infiltration in glioma, especially M2 macrophage, and verified by RT­PCR, Western blotting, and immunohistochemistry using our clinical glioma samples. *MLLT11* had a highly negative correlation with immune checkpoint inhibitor (ICI) genes including *PDCD1*, *PD-L1*, *TIM3(HAVCR2)*, and *PD‐L2 (PDCD1LG2)*.

**Conclusion:**

*MLLT11* plays a crucial role in the progression of glioma and has the potential to be a new prognostic marker for glioma.

## Introduction

Glioma, divided into lower-grade glioma (WHO grades 2 and 3) and high-grade glioma (WHO grade 4), is one of the most common intracranial malignant tumors, and its annual incidence rate is about 3–6.4/100,000. Among them, glioblastoma (GBM) contributes to the worst prognosis, with a median survival time of approximately 12–15 months even after standard treatment of surgery, radiotherapy, and chemotherapy (1, 2). Nowadays, glioma molecular characteristics, such as the status of isocitrate dehydrogenase (IDH) mutation, methylation of O6-methylguanine-DNA methyltransferase (MGMT), and 1p/19q codeletion status, complement traditional therapy, and tumor-treating fields (TTFs) have been identified as important indicators for glioma classification and outcome prediction (1, 3). New therapies such as molecular targeting treatment and immunotherapy are enrolled in the treatment strategy, but only a few are actually able to improve clinical outcomes. The potential reason is that glioma tends to be a multi-gene and multi-step genetic heterogeneous disease, and tumor heterogeneity may probably be the root of treatment failure (4, 5). Recently, it has been found that GBM can be further divided into four different subtypes, pro-neural (TCGA-PN), neural (TCGA-NE), classical (TCGA-CL), and mesenchymal (TCGA-MES) (6, 7), based on their molecular feature. Neftel et al. (8) confirmed the existence of four different cell subtypes in GBM at the single-cell level, including neural progenitor-like (NPC-like), oligodendrocyte progenitor-like (OPC-like), astrocyte-like (AC-like), and mesenchymal-like (MES-like) states, and relate them to the corresponding bulk subtypes. In addition, the tumor immune microenvironment plays a pivotal role in tumor aggression. The tumor and its microenvironment influence each other during the tumor growth through cellular signaling of molecular or infiltrated immune cells. GBM can induce the activation of a variety of immune cell types, such as tumor-infiltrating macrophages, which produce a large number of cytokines, growth factors, and interleukin, so as to produce a suitable tumor microenvironment (TME) and promote the growth and proliferation of glioma cells (9). Thus, an in-depth understanding of the key molecules and mechanisms is of great significance for the diagnosis and treatment of glioma.

The*MLLT11* gene, located on chromosome 1q21, encodes a protein with a molecular weight of ∼9 kDa and consists of 270 amino acids (10). Regarding the function of*MLLT11* as an “oncogene” signature in hematologic diseases (11), studies are currently mainly focused on hematologic disorders. The expression of*MLLT11* markedly increased in several hematologic disorders, including lymphocytic leukemia, lymphoma, and myelodysplastic syndrome (12, 13). Although the specific function of*MLLT11* is not well defined, it exhibits proapoptotic functions in some solid tumors, such as ovarian and liver cancer (10, 14, 15).

At present, it has been shown that there is a correlation between the level of*MLLT11* expression and the degree of cell differentiation (16–18). In the central nervous system (CNS),*MLLT11* expression is gradually upregulated as neural stem cells (NSCs) differentiate into neurons, and its high expression promotes neuronal differentiation and maturation (16, 19). Moreover,*MLLT11* also plays an important role in tumor cell and immune cell interaction in the TME (20). Considering the*MLLT11* function in various tumors and to reveal its function in glioma, we explored its expression pattern and clinicopathological significance that can offer new insights for glioma treatment.

In this study, we analyzed the expression profile of*MLLT11* in gliomas. Large-scale bioinformatic analysis, enrolling both bulk and single-cell data, was performed by downloading gene expression data from known databases. Then,*MLLT11* expression in tumor samples and normal brain tissues was confirmed by real-time quantitative polymerase chain reaction (qPCR), Western blotting, and immunohistochemistry (IHC) in our institute. Moreover, we evaluated the prognostic value of*MLLT11* in the treatment for gliomas systematically and comprehensively. We found that among gliomas, there were distinct differences in*MLLT11* expression among different grades of gliomas, and there was also a clear propensity for*MLLT11* expression among the different subtypes. In addition, we explored the relationship between*MLLT11* and immune infiltration and found that, with increasing malignancy, the expression level of*MLLT11* showed a decreasing trend, and*MLLT11* expression was negatively correlated with M2-type macrophages in the TME. Furthermore, by qPCR, Western blotting, and IHC, we found that both mRNA and protein levels of M2-type macrophage-specific markers were obviously increased with higher glioma pathological grade. These results suggest that in high-grade gliomas, along with downregulated*MLLT11* expression, more M2-type macrophages are recruited into the TME to promote tumor growth. Based on these data, we conclude that*MLLT11* is a potential prognostic biological marker and may probably be a clinical therapeutic target for glioma patients.

## Materials and methods

### Dataset and data processing

We collected expression data and corresponding metadata from lower-grade glioma (LGG) and GBM samples in TCGA, CGGA, and GEO databases. A total of 697 samples from TCGA were downloaded from The University of California Santa Cruz (UCSC) Xena (https://xenabrowser.net/), including LGG and GBM. In addition, 1,319 samples were downloaded from the CGGA website (http://www.cgga.org.cn/). CGGA samples included mRNAseq_693, mRNAseq_325, and CGGA_array datasets. Furthermore, 444 bulk samples were collected from GEO datasets, including GSE43378, GSE16011, GSE74187, and GSE83300. A total of 3,589 cells from four primary GBM patients’ single-cell RNA sequencing (scRNA-seq) data according to Smart-seq2 protocol, GSE84465, were normalized following Seurat (v3.1.1) pipeline (21, 22). In this study, 105 normal brain cortex samples from GTEx databases were used for comparisons (http://commonfund.nih.gov/GTEx/). All bulk RNA-seq count data were normalized into transcripts per kilobase million (TPM). In addition, we also collected posttraumatic normal brain tissue (n = 9) and glioma samples (n = 27) from the Department of Neurosurgery in Xiangya Hospital, Central South University, including nine cases of grade 2, 3, and 4 gliomas, respectively. This study was approved by the medical ethics committee of Xiangya Hospital, Central South University.

### Group division and survival analysis

The patients were divided into*MLLT11* ^high^ and*MLLT11* ^low^ groups according to*MLLT11* expression and the survival data by the cutoff calculated by the maximally selected rank statistics. The Kaplan–Meier survival curve was depicted to estimate survival distribution by R packages named *survival* and *survminer*. The log-rank test was used to assess statistical significance between groups. The coxph function in R package survival was used for Cox regression analysis. The receiver operating characteristic (ROC) curve was generated to assess the accuracy of the model with the R package *pROC* (23).

### Differential gene identification and enrichment analysis

The differentially expressed genes (DEGs) were identified in each dataset through using the R package *limma* for normalized data false discovery rate (FDR) <0.05 and |log2 [fold change (FC)]|>1.5). Then, with the R package *clusterProfiler* (24), the pathway enrichment analysis was performed for the up-expressed and the down-expressed DEGs. The functional and pathway enrichment analysis includes Gene Ontology (GO) and Kyoto Encyclopedia of Genes and Genomes (KEGG) pathway. The KEGG gene signatures were obtained from the Molecular Signatures Database v6.2 version (MSigDB, https://www.gsea-msigdb.org/gsea/msigdb). The pathway was scored using the single-sample gene set enrichment analysis (ssGSEA) (25), as implemented in the *GSVA* R package (26). Then, principal component analysis (PCA) was used for dimensionality reduction of the pathway score matrix.

### Immune infiltration analysis

CIBERSORT deconvolution algorithm was introduced, referring to the LM22 set (27) and the normalized signature matrices (28), to analyze the relative abundance of infiltrating immune cells. The association analysis and plots were conducted with the utilization of the R package *ggstatsplot*.

### qPCR

Total RNA of the collected glioma tissues and normal tissue samples was extracted by TRIzol kit (Accurate Biology, China, AG21101) and RNA purification kit (Thermo Scientific, K0731), reverse transcribed to cDNA by RT-qPCR kit (Thermo Scientific, K16225). Amplification was performed according to the SYBR green (Bio-Rad, #1725274) method with three replicate wells per sample in a total reaction system of 20 μl. PCR reaction conditions (ABI 7300 Real-Time System) were as follows: 95°C for 15 min, 40 cycles of amplification at 95°C for 15 s, 60°C for 1 min. β-Actin was used as an internal reference and 2**^-ΔΔCt^** formula was used to calculate the relative expression of the targeted gene. Targeted gene primer sequences were as follows:*MLLT11*: (forward) 5’-GTAGCCAGTACAGTTCCTTTCT-3’, (reverse) 5’-AAGTTGAAGGTGCTGTACTCAA-3’; *ARG1*: (forward) 5’-GGACCTGCCCTTTGCTGACATC-3’, (reverse) 5’-TCTTCTTGACTTCTGCCACCTTGC-3’; *CD206*: (forward) 5’-TCCGACCCTTCCTTGACTAATCCTC-3’, (reverse) 5’-AGTATGTCTCCGCTTCATGCCATTG-3’; *IL-10*: (forward) 5’-GTTGTTAAAGGAGTCCTTGCTG-3’, (reverse) 5’-TTCACAGGGAAGAAATCGATGA-3’; *CD163*: (forward) 5’-ATCAACCCTGCATCTTTAGACA-3’, (reverse) 5’-CTTGTTGTCACATGTGATCCAG-3’; *CD115*: (forward) 5’-GTCCTGAAGGTGGCTGTGAAGATG-3’, (reverse) 5’-GCTCCCAGAAGGTTGACGATGTTC-3’; PDGFβ: (forward) 5’-TCTCTGCTGCTACCTGCGTCTG-3’, (reverse) 5’-AAGGAGCGGATCGAGTGGTCAC-3’.

### Western blotting

Clinical glioma samples and normal brain tissue samples were collected, and total protein was extracted. The concentration of protein was determined by the bicinchonininc acid (BCA) method and calculating the loading capacity. Boiled at 100°C for 5 min and stored at -80°C for further use. The protein sample (100 ng) was separated by 15%, 10% sodium dodecyl sulfate polyacrylamide gel electrophoresis (SDS-PAGE), and the separated protein bands were transferred onto the polyvinylidene fluoride (PVDF) membrane. The membrane was blocked in 5% nonfat milk for 2 h at room temperature and washed for 10 min with phosphate buffer solution with 0.05% Tween 20 (PBST) (three times), then incubated overnight at 4°C with*MLLT11* I antibody solution (1:2,000, Abcam, ab109016) and arginase-1 (*ARG1*) antibody (1:1,000, Proteintech, 16001-1-AP) that had been diluted in PBST and then washed with PBS for 10 min (three times). The membrane was incubated in the antibody II solution (Goat Anti-Rabbit IgG, 1:5,000, Proteintech) for 1 h at room temperature and washed with PBST for 10 min (three times). The protein bands stained by the antibody were visualized using enhanced chemiluminescence (WesternBright Sirius, Advansta, 200425-03), and β-actin was used as an internal reference followed by exposure to X−ray films (Bio-Rad). Relative protein expressions were analyzed from band intensities using ImageJ software.

### Immunohistochemistry

Glioma specimens of different grades and normal brain tissue were fixed with formalin, embedded in paraffin, and then made into 4-µm sections. Sections were dewaxed and rehydrated pretreated for antigen retrieval in citrate buffer and quenched for endogenous peroxidase with 3% hydrogen peroxide (H_2_O_2_). Nonspecific antigenic sites were blocked with 10% normal goat serum, and sections were incubated overnight with*MLLT11* I antibody (1:100, Abcam, ab109016) and *CD163* I antibody (1:500, CST, #93498) at 4°C. These were then incubated with secondary antibody (goat anti-rabbit IgG, 1:5,000, Proteintech) and stained with diaminobenzidine tetrahydrochloride (DAB) and hematoxylin. IHC images were acquired, and the score of*MLLT11* and *CD163* protein expression was calculated by image processing software ImageJ.

### Statistical analysis

All bioinformatic statistical analyses in this study were performed using R version 4.1.0 (The R Foundation for Statistical Computing, http://www.rproject.org/). The Wilcoxon rank-sum test was used to determine the expression levels of*MLLT11* with regard to pathological characteristics. The survival probability was determined using Kaplan–Meier survival curves by log-rank test. The Pearson correlation was applied to evaluate the relationship between*MLLT11* expression and immune cell infiltration and immune checkpoints. The heatmap was drawn with the R package *pheatmap*. All statistical tests were two-sided. The p < 0.05 was considered statistically significant.

## Results

### Clinical and molecular characteristics of*MLLT11* in gliomas

**Figure 1:**
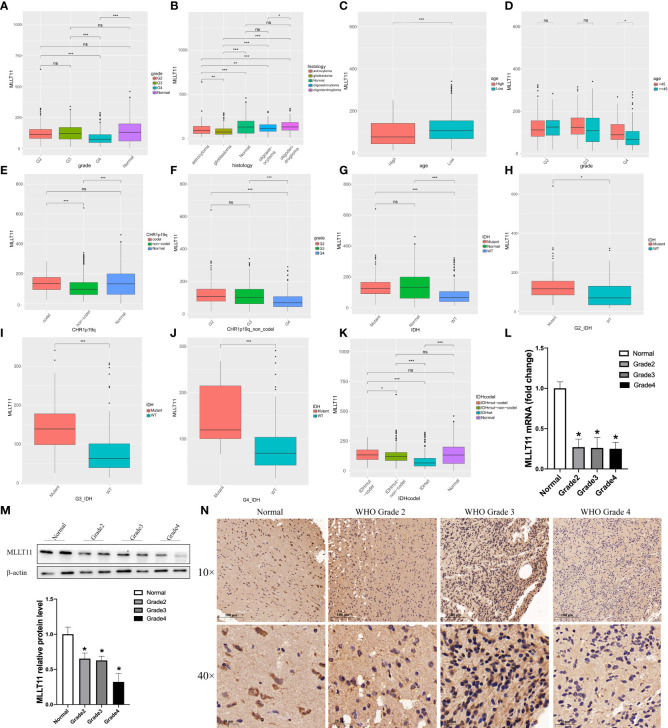
Clinical and molecular characteristics of MLLT11 in gliomas. **(A, B)** The expression of MLLT11 in different WHO pathological grades. **(C, D)** The expression of MLLT11 stratified by age and WHO pathological grades. **(E, F)** The expression of MLLT11 in 1p19q codeletion and non-codeletion glioma. **(G–J)** The expression of MLLT11 in IDH mutant and wild type and stratified by age and different WHO pathological grades. **(K)** The expression of MLLT11 in IDH mutation combined with 1p/19q codeletion and IDH mutation combined with 1p/19q non-codeletion. **(L, M)** Representative images of qPCR and Western blotting for MLLT11 in normal brain tissue and different tumor grades. **(N)** Representative images of IHC staining for MLLT11 in normal brain tissue and different WHO pathological grades of glioma. *p < 0.05, **p < 0.01, ***p < 0.001; ns, no statistics. G2, Grade 2; G3, Grade 3; G4, Grade 4; IDH, isocitrate dehydrogenase; WT, wildtype.

We first compared the*MLLT11* expression among LGG, GBM, and normal brain cortex in TCGA and GTEX dataset and found no significant difference between normal brain and WHO grade 2 and WHO grade 3 gliomas, but the expression of*MLLT11* in GBM is the lowest among the four histological groups. Then, the exploration of*MLLT11* expression in glioma histology revealed that GBM showed the lowest expression, followed by astrocytoma, and no difference between normal brain, oligoastrocytoma, and oligodendroglioma (**Figures 1A, B** **)**. According to median age, patients were divided into high and low age subgroups, and there were significant differences in the expression of*MLLT11* between the high age subgroup and the low age subgroup in TCGA datasets (**Figure 1C**). Meanwhile, no significant*MLLT11* expression difference was found in LGG between the young and old groups except for GBM (**Figure 1D**). The same result is also seen in CGGA dataset (**Supplementary Figures 1A, B**). Furthermore, the*MLLT11* expression difference can also be analyzed in different WHO pathological grades in the high age subgroup and the low age subgroup. The result shows that in both high-age and low-age groups, the expression level of*MLLT11* has a significant downward trend with the increase of tumor grade (**Supplementary Figures 1C, D**). Of note, in the CGGA dataset and TCGA dataset, no significant difference was found between men and women for*MLLT11* expression in different WHO pathological grades (**Supplementary Figures 1E–G**).

In recent years, with the rapid development of the molecular pathology of glioma, a series of molecular markers, such as IDH mutation, 1p/19q codeletion, and MGMT methylation status, have been considered to be related to the malignancy of glioma and patient prognosis. Therefore, these clinical-related molecular biomarkers were also explored in this study. We found that there was a significant difference in the expression of*MLLT11* between 1p19q codeletion and non-codeletion status in TCGA datasets. Compared with 1p19q non-codeletion, the expression of*MLLT11* in 1p19q codeletion was higher (**Figure 1E**), and the same result was found in LGG (**Supplementary Figures 1H, I**). In patients with non-codeletion, the expression of*MLLT11* gradually decreased with the increase of tumor grade (**Figure 1F**). Furthermore, the expression level of*MLLT11* was significantly different between IDH mutation and non-mutation, which was validated in CGGA and TCGA datasets. The expression of*MLLT11* in the IDH mutant subtype, similar to the normal brain, was significantly higher than that of IDH wild-type glioma, and the difference was more obvious in GBM (**Figures 1G–J**). A similar result was found in CGGA_325, CGGA_693, and CGGA_array datasets (**Supplementary Figures 1J–O**). In addition, we also found that compared with IDH mutation or 1p/19q codeletion alone, the expression of*MLLT11* with IDH mutation combined with 1p/19q codeletion was the highest (**Figure 1K**), suggesting that the prognostic value of*MLLT11* expression is consistent with the known IDH mutation combined with 1p/19q codeletion.

qPCR, Western blotting, and IHC staining were used to detect the mRNA transcription level and protein expression of*MLLT11* in glioma samples of different grades (n = 27) and normal brain tissues (n = 9) from our institution. The clinical characteristics and molecular pathology of these patients are shown in **Supplementary Table 1**. qPCR results showed that the expression level of*MLLT11* in tumor tissues was significantly lower than that in normal brain tissues (p < 0.05), but no significant difference was found between different grades of glioma (**Figure 1L**). Furthermore, Western blotting and IHC results confirmed that the expression of*MLLT11* was higher in normal brain tissues and LGG when compared with that of GBM tissues (**Figures 1M, N** **)**. Moreover, glioma patients with higher*MLLT11* expression level experienced favorable outcomes among the glioma patients in the above analysis. Based on the above information, we infer that*MLLT11* decreased significantly in GBM and may play an important role in invasive processes of gliomas.

### MLLT11 expression level shows a subtype preference in glioblastoma

**Figure 2:**
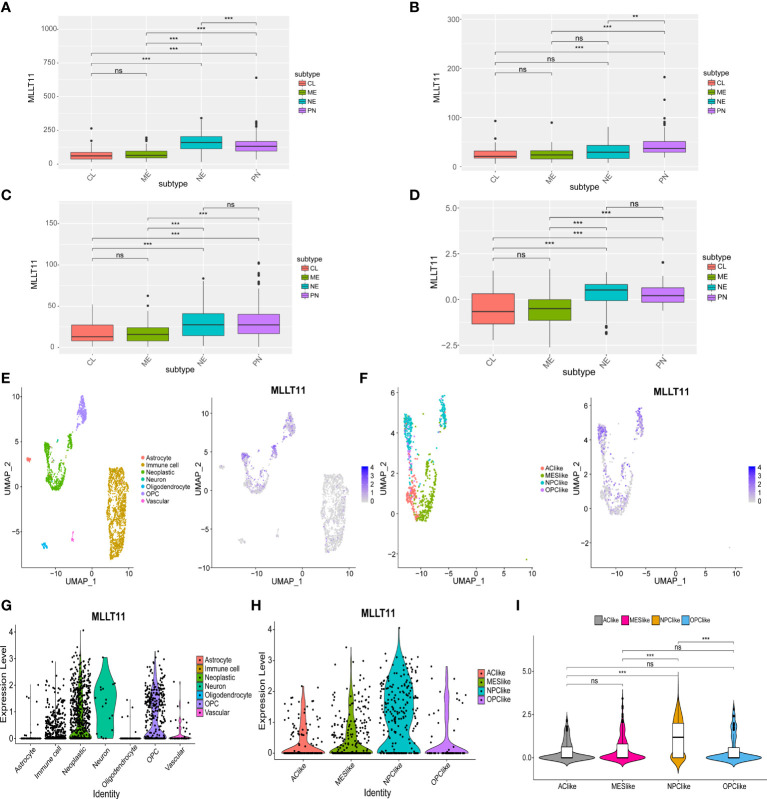
The expression of MLLT11 in different glioma subtypes. **(A–D)** MLLT11 expression level in different molecular subtypes in TCGA, CGGA_325, CGGA_691, and CGGA_array datasets based on bulk expression. **(E–I)** ScRNA-seq results for MLLT11 expression in GBM. **(E)** The cells were categorized into eight clusters (left). Blue scatter plots represent MLLT11 expression distribution in eight clusters (right). **(F)** Tumor cells were extracted and categorized into four different cell clusters based on the scRNA expression level (left), and MLLT11 expression is shown in blue scatter spots (right). **(G, H)** The expression levels of MLLT11 in the eight cell clusters and four molecular subtypes were shown by violin plot. **(I)** MLLT11 was mainly expressed in NPC-like subtypes compared with the other three subtypes. **p < 0.01, ***p < 0.001; ns, no statistics.

Currently, during clinical diagnosis and treatment, molecular subclasses in glioma provide new insights into predicting patient prognosis (6), and studies based on bulk expression in TCGA suggest that GBM can be classified into four subtypes, namely, classical (TCGA-CL), mesenchymal (TCGA-ME), pro-neural (TCGA-PN), and neural (TCGA-NE), of which the PN subtype has a relatively good prognosis, but the CL and ME subtypes are more aggressive and have a poor prognosis (7, 29). In this study, we detected*MLLT11* expression in LGG, GBM samples, and normal tissues from TCGA and GTEx datasets and found that increased*MLLT11* expression was associated with the NE and PN subtypes in TCGA and CGGA datasets (**Figures 2A–D**).

To clarify the role of*MLLT11* in glioma, we further analyzed*MLLT11* expression in gliomas from single-cell dimension. Seven clusters of cells were identified from four glioma samples, mainly including astrocytes, oligodendrocytes, oligodendrocyte precursor cells (OPCs), neoplastic cells, neurons, vascular endothelial cells, and immune cells (**Figure 2E**). The expression of*MLLT11* was mainly concentrated in neoplastic cells, immune cells, and OPCs (**Figure 2F**). In addition, scRNA-seq clustering was carried out according to the four types proposed in GBM by Neftel et al. (8) in 2019, mainly including four cell clusters, AC-like, NPC-like, OPC-like, and MES-like, in which TCGA-PN subtype corresponds to NPC-like cellular state (8). Our results showed that*MLLT11* was mainly expressed in NPC-like cells, which was significantly different from the other three clusters of cell subtypes (**Figures 2G–I**). This is consistent with the expression of bulk tumor sequencing, that is,*MLLT11* is mainly upregulated in TCGA-NE and TCGA-PN. It is suggested that*MLLT11* can be used as a marker of GBM subtype.

### The expression level of*MLLT11* refers to the prognosis of the glioma patient

**Figure 3:**
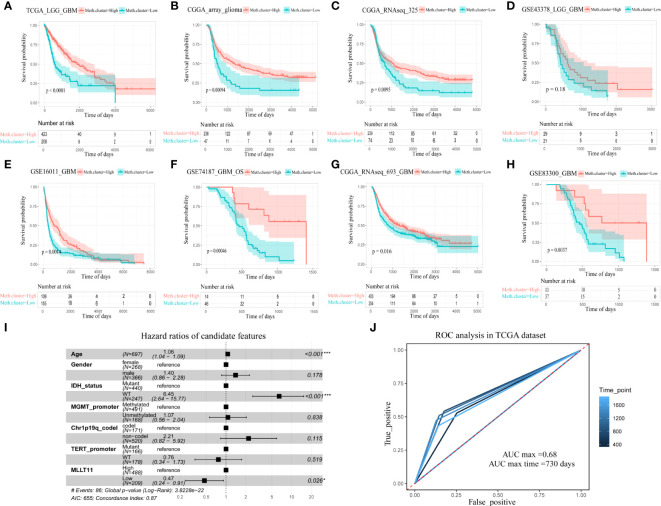
The relationship between MLLT11 expression and survival for glioma. **(A–H)** Kaplan–Meier plots of MLLT11 in TCGA and CGGA datasets, **(A)** TCGA_LGG_GBM, **(B)** CGGA_array, **(C)** CGGA_325, **(D)** GSE43378, **(E)** GSE16011, **(F)** GSE74187, **(G)** CGGA_693, and **(H)** GSE82009. **(I)** The forest diagram represents the multifactor Cox regression analysis in which the variables include MLLT11, gender, age, IDH mutation status, 1p19q deletion status, MGMT methylation, and TERT promoter status. **(J)** ROC curve analysis of the model in pan-glioma cohort in TCGA dataset. *p < 0.05, **p < ***p < 0.001.

From the above bioinformatic analysis result, we conclude that the expression of*MLLT11* has significant correlation with glioma malignancy, i.e., in gliomas,*MLLT11* expression tends to decrease with increasing tumor malignancy. To further test and verify the reliability of this result, we evaluated the prognostic value of*MLLT11* in eight datasets from TCGA, CGGA, and GEO. In these datasets, patients were divided into low-expression subgroup and high-expression subgroup, and survival analysis was performed using log-rank test analysis. The results revealed that, in TCGA (p < 0.0001), CGGA_array (p < 0.001), and CGGA_RNAseq_325 datasets (p < 0.01), patients (LGG and GBM) with a higher expression of*MLLT11* showed significantly better prognosis than that of the low-expression subgroup (**Figures 3A–C**). In the GSE43378 dataset, although no obvious statistical significance in the survival analysis was found between the*MLLT11* high- and low-expression groups, there was still a similar trend between them (p = 0.16) (**Figure 3D**). Also, among GBM patients, we also find that patients with a high expression of*MLLT11* show better prognosis in CGGA_RNAseq_693 dataset (p < 0.05), GSE16011 dataset (p < 0.01), GSE74187 dataset (p < 0.001), and GSE83300 dataset (p < 0.01) (**Figures 3E–H**).

To explore the influence on the survival of glioma patients, we evaluated the prognostic value of*MLLT11* in TCGA dataset cohorts. We applied the multivariate Cox regression analysis to investigate the prognostic power of the signature, and the result revealed that the expression of*MLLT11* hazard ratios (HR) = 0.47, p < 0.026) is an independent prognostic biomarker for glioma patients after adjusting for age, gender, IDH, MGMT promoter, Telomerase reverse transcriptase (TERT) promoter, and 1p19q codeletion status (**Figure 3I**). Moreover, ROC curve analysis in TCGA dataset was used to comprehensively assess the sensitivity and specificity of the model. The area under the curve (AUC) of*MLLT11* was 68% in the pan-glioma cohort (**Figure 3J**).

### Differentially expressed genes and functional enrichment analysis between the*MLLT11* high- and low‐expression groups

**Figure 4:**
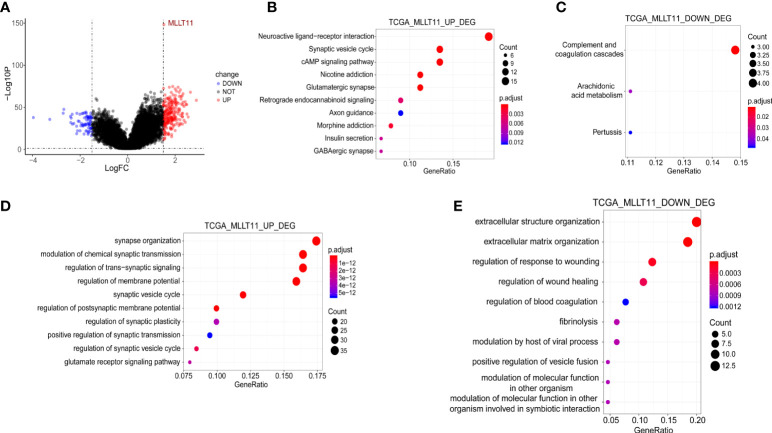
Functional enrichment analysis between the MLLT11 high- and low‐expression groups in gliomas. **(A)** Differential expression genes (DEGs). **(B, C)** KEGG enrichment analysis of the upregulated genes in the high MLLT11 subgroup and the downregulated genes in the high MLLT11 subgroup. **(D, E)** GO analysis of the upregulated genes in the high MLLT11 subgroup and the downregulated genes in the high MLLT11 subgroup.

We analyzed DEGs between the groups with low and high*MLLT11* expression in TCGA. By using the criteria |log FC| >1.5 and FDR <0.05, we obtained 301 DEGs (230 upregulated and 71 downregulated) (**Figure 4A**). The KEGG enrichment analysis indicated that the*MLLT11* upregulated DEGs were involved in pathways related to neuroactive ligand–receptor interaction, synaptic vesicle cycle, cAMP signaling pathway, glutamatergic synapse, and GABAergic synapse (**Figure 4B**), while the*MLLT11* downregulated DEGs were involved in pathways related to complement and coagulation cascades and arachidonic acid metabolism (**Figure 4C**). Similarly, in the CGGA_325 dataset and CGGA_array dataset, the*MLLT11* upregulated genes are also mainly enriched in GABAergic synapse, retrograde endocannabinoid signaling, synaptic vesicle cycle, and glutamatergic synapse (**Supplementary Figures S2B**, **S3B**). The*MLLT11* downregulated genes were mainly enriched in the pathway of extracellular matrix (ECM)–receptor interaction, protein digestion and absorption, and complement and coagulation cascades (**Supplementary Figure 2C**). In the biological process category, in TCGA dataset,*MLLT11* upregulated DEG-enriched GO terms were identified, mainly involved in the pathway of the development and regulation of the synapse structure, regulation of membrane potential, and postsynaptic membrane potential (**Figure 4D**). MLLT11 downregulated DEGs were mainly enriched in the pathway of ECM and extracellular structure organization and regulation of response to wounding and regulation of wound healing (**Figure 4E**). The same results were also found in CGGA datasets (**Supplementary Figures 2D, E**, **3C, D**). Based on these analyses,*MLLT11* may influence the glioma microenvironment *via* ECM organization and might promote the synapse information to influence the progression of glioma.

### Principal component analysis and*MLLT11*-related biological process

**Figure 5:**
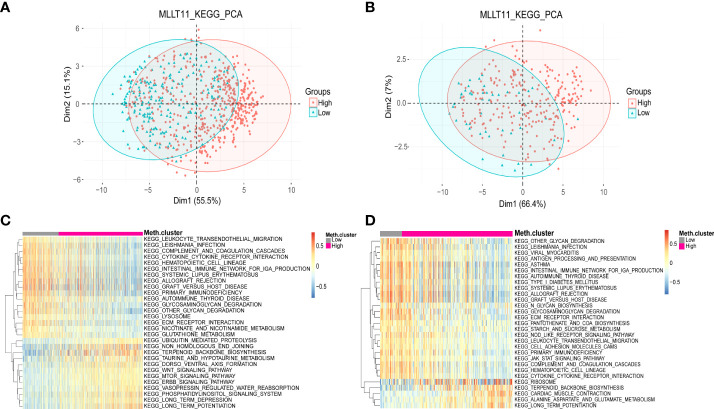
Distinct metabolism status in patients with MLLT11 low expression and high expression in glioma. **(A, B)** PCA between the low-expression and high-expression groups based on total DEGs in TCGA and CGGA dataset. **(C, D)** KEGG pathway enrichment analyses of DEGs in MLLT11 low-expression and high-expression groups in TCGA and CGGA dataset.

Based on the results of clinicopathological characteristics and survival analysis, we deduced that*MLLT11* might play an essential biological function in glioma initiation and progression. To further investigate the biological process associated with*MLLT11* expression, we performed PCA to investigate differences in metabolism status between the low-expression subgroup and high-expression subgroup based on the expression of*MLLT11*. PCA results showed that the contribution of PCA1(Dim1) and PCA2(Dim2) to the total variance accounted for 55.5% and 15.1%, respectively, in TCGA dataset (**Figure 5A**). Similarly, in the CGGA dataset, Dim1 and Dim2 account for 66.4% and 7% to the total variance, respectively (**Figure 5B**). Furthermore, we performed KEGG enrichment analyses of the DEGs to explore the difference of metabolism status between the*MLLT11* low-expression and high-expression subgroups in TCGA and CGGA. We found that, in the low-expression subgroup, the DEGs were significantly involved in multiple tumor- and immune-related pathways, such as leukocyte trans-endothelial migration, cytokine–cytokine receptor interaction, intestinal immune network for IgA production, graft-versus-host disease, primary immunodeficiency, autoimmune thyroid disease, glycosaminoglycan degradation, lysosome, ECM–receptor interaction, and glutathione metabolism (**Figures 5C, D** **)**. However, in the*MLLT11* low-expression subgroup, ubiquitin-mediated proteolysis, taurine and hypotaurine metabolism, WNT signaling pathway, mammalian/mechanistic target of rapamycin (mTOR) signaling pathway, including erb-B1 (EGFR, HER1),erb-B2(HER2)、erb-B3(HER3) and erb-B4(HER4) (ERBB) signaling pathway, and phosphatidylinositol signaling system were enriched. These results have occurred similarly in other datasets in the CGGA (**Supplementary Figures 4A–D**). These suggest that the aggressiveness of glioma is associated with an abnormal immune status.

### Relationships between*MLLT11* expression and immune cells and immune checkpoint markers

**Figure 6:**
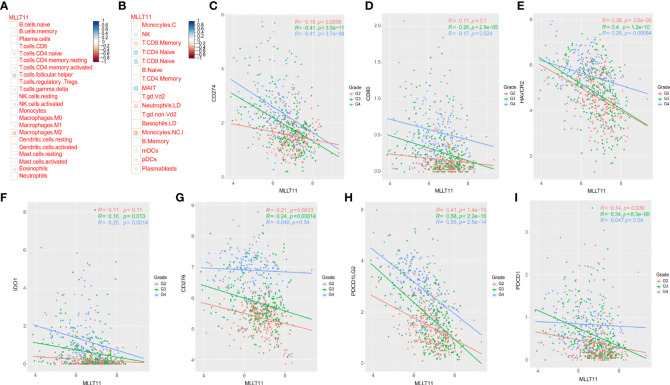
Relationships between MLLT11 expression and immune cells and immune checkpoint markers. **(A, B)** Correlation analysis of MLLT11 with 22 immune cells in TCGA dataset. **(C–I)** Correlation between MLLT11 and seven immune checkpoints in TCGA dataset. **(C)** CD274, **(D)** CD80, **(E)** HAVCR2, **(F)** IDO1, **(G)** CD276, **(H)** PDCD1LG2, **(I)** PDCD1.

**Figure 7:**
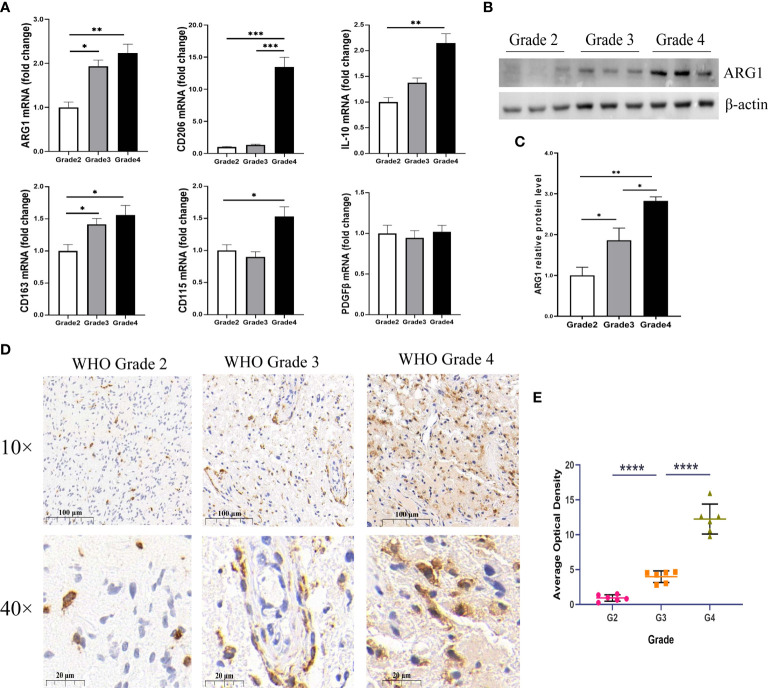
Expression of M2 macrophage markers in different WHO pathological grades of glioma. **(A)** The mRNA expression levels of M2-type macrophage-specific markers, including ARG1, CD206, IL-10, CD163, CD115, and PDGFβ in different grades of glioma samples (n = 24). **(B)** Representative Western blotting images of ARG1 and **(C)** bar charts of normalized protein expression levels of ARG1 in different grades of gliomas samples (n = 18). **(D)** Representative images of immunohistochemical (IHC) staining for CD163 in different grades of gliomas (n = 18). **(E)** Quantification of CD163 IHC staining in different pathological grades of gliomas (n = 18). *p < 0.05, **p < 0.01, ***p < 0.001, and ****p < 0.0001.

To investigate the mechanism by which high expression of*MLLT11* contributes to a better prognosis, we explored the correlation between*MLLT11* expression and immune infiltrating levels of 22 immune cells by CIBERSORT algorithm, including B cells, T cells, Natural killer (NK) cells, monocytes, macrophages, dendritic cells, mast cells, eosinophils, and neutrophils. Pearson correlation analysis was used to identify immune cells that strongly associated with*MLLT11* expression (|r| > 0.4, p < 0.05) in the CGGA and TCGA sequencing datasets. Results in TCGA dataset show that the expression of*MLLT11* was positively correlated with the infiltration level of naive CD4^+^ T cells and CD8^+^ T cells and negatively correlated with the infiltration level of macrophages, especially M2 macrophages (**Figures 6A, B** **)**. Similar results were found in CGGA array and CGGA 325 dataset (**Supplementary Figures 5A, B**). Furthermore, by qPCR, IHC, and Western blotting, in different pathological grades of glioma tissues, we found a significant increase in the expression levels of M2-type macrophage-specific markers with increasing tumor grade, including *CD206*, *CD163*, *ARG1*, *CD115*, and *IL-10* (**Figure 7**). This may be that in the process of tumor progression, with the decrease of*MLLT11* expression level and the increase of immune infiltration level, the generated M2 immune cells might promote the aggressive process.

Recently, immunotherapy has dramatically advanced in the treatment of cancer. Considering the importance of immune checkpoint molecules for the immune response and immunotherapy, several common immune checkpoint members were enrolled in our analysis and assessed the correlation between*MLLT11* and these immune checkpoints, such as HAVCR2 (TIM-3), CD274 (*PD-L1*), *CD276 (B7-H3)*, *CD80*, *PDCD1*LG2 (PD-L2), *PDCD1* (PD-1), and IDO1. Pearson correlation analysis was performed to explore the relationship between the*MLLT11* expression level and seven immune checkpoints in CGGA dataset and TCGA dataset. In TCGA datasets, among WHO grade 3 glioma,*MLLT11* showed a highly negative correlation with PD-1 (r = -0.34, p = 8.3 × 10^-28^), *PD-L1* (r = -0.41, p = 3.5 × 10^-11^), *TIM3(HAVCR2)* (r = -0.4, p = 1.2 × 10^-11^), and PD‐L2 (*PDCD1*LG2) (r = -0.58, p < 2.2 × 10^-16^) (**Figure 6**). Similarly, a strong negative correlation was found between*MLLT11* and *PD-L1* (r = -0.41, p = 3.7 × 10^-8^), *TIM3(HAVCR2)* (r = -0.26 p = 8.4 × 10^-4^), and PD‐L2(*PDCD1*LG2) (r = -0.55, p = 2.5 × 10^-14^) in GBM (**Figure 6**). A similar trend can be seen in the CGGA_325 and CGGA_array datasets (**Supplementary Figures 6A–G**, **7A–G**). These findings indicate that*MLLT11* may have a critical role in the immune response; especially when*MLLT11* expression is low, immunotherapy may be more effective.

## Discussion

Gliomas account for 80% of all malignant brain tumors, and GBMs occupy about 50% of gliomas. Due to the existence of inter- and intra-glioma heterogeneity, despite the standard treatment of precision surgery, combination of radiotherapy and chemotherapy, and even TTFs, the 5-year survival rate for GBM remains unsatisfactory. Thus, novel therapeutic approaches are needed urgently. However, the origin and progression of glioma are extremely complex processes with multiple steps, and multiple regulators have been confirmed to be involved in the regulation of this process; therefore, exploring and identifying suitable regulators are feasible approaches for the treatment of glioma.

The*MLLT11* gene is primarily responsible for encoding a 9-kDa transmembrane protein and highly expressed in some cancer cell lines and normal hematopoietic tissues (30, 31). Elevated*MLLT11* expression was associated with poor prognosis in several human cancers, such as pediatric acute myeloid leukemia, ovarian cancer, and gastrointestinal malignancy (32, 33). However, in the nervous system, the opposite is true. During embryonic development, studies have shown that*MLLT11* plays an important role in neuronal differentiation and maintenance. Lin et al. (19) found that during the differentiation of neural stem/precursor cells into neurons, the expression of*MLLT11* was significantly upregulated. This indicates that*MLLT11* may play an essential role in neuronal differentiation during CNS development. Our results found that*MLLT11* is highly expressed in normal brain tissues and decreased with the increase of tumor grade. Based on these pieces of evidence, in the CNS, we infer that*MLLT11* is at least involved in promoting differentiation and inhibiting cancer development.

In analyzing the age effect, we found a significant statistical difference in the expression of*MLLT11* between the young and old subgroups. In the old subgroup, the expression of*MLLT11* was low, and *vice versa*. Further analysis stratified by tumor grade indicated that there was no statistical difference in the expression of*MLLT11* between the young and old subgroups in LGG, but in GBM,*MLLT11* expression in the old subgroup was significantly lower than that in the young subgroup. Based on these pieces of evidence, considering that the incidence of GBM is often in elderly patients, we speculated that*MLLT11* may be a gene promoting differentiation and a tumor-suppressive effect, and*MLLT11* may play a key role in the progression of glioma. In addition, 1p19q codeletion and IDH mutation status are of great significance in predicting the prognosis of glioma. Moreover, 1p19q codeletion and IDH mutation suggest a better prognosis. In our analysis, compared with patients with 1p19q codeletion and IDH mutation alone,*MLLT11* expression is higher in the combination of the two, indicating that the expression pattern of*MLLT11* has a similar role in predicting the prognosis of glioma patients as that of 1p19q deletion and IDH mutation.

We further analyzed the expression of*MLLT11* in the RNA-seq data from TCGA and CGGA. It was firmly established that the expression of*MLLT11* was obviously upregulated in TCGA-PN phenotype compared with TCGA-MES. Furthermore, from an scRNA-seq perspective, we deeply analyzed*MLLT11* expression differences in different cell clusters, and the results demonstrated that*MLLT11* was mainly expressed in neoplastic cells, immune cells, and OPC clusters. The*MLLT11* expression in tumor cells was further analyzed to explore the expression distribution in four subtypes of GBM based on single-cell sequencing proposed by Neftel et al. (8). The scRNA-seq analysis suggested that the*MLLT11* expression level in NPC-like subtypes was significantly higher than that of the other three subtypes, AC-like, OPC-like, and MES-like. The bulk PN subtype corresponded to the NPC-like cell subtype, with both subtypes referring to better prognosis in their own classification. Thus, the expression distribution of*MLLT11* in the bulk sample was consistent with that at single-cell sequencing level. Furthermore, survival analysis based on independent datasets from TCGA and CGGA datasets found that high levels of*MLLT11* expression tend to predict better outcomes. These results lead us to believe that*MLLT11* has an important predictive value for glioma prognosis.

In order to further explain the clinical prognostic role of*MLLT11*, we performed KEGG and GO enrichment analysis on the DEGs. The upregulated DEGs of*MLLT11* were mainly enriched in neuroactive ligand–receptor interaction, synapse organization, synaptic vesicle cycle, and the regulation of synapse-related signals, as well as glutamatergic synapse-related signaling pathway. As we all know, epilepsy is a common symptom in patients with glioma and the combination of antiepileptic drugs plays an important role in the treatment of glioma (34, 35). Venkataramani et al. (36) revealed a direct link between neurons and glioma cells through different glioma disease models and clinical tumor specimens and finally confirmed that functional chemical synapses exist between presynaptic neurons and postsynaptic glioma cells. Also known as neuron–glioma synapses (NGSs), these synaptic-like structures play a “bidirectional switch” role in gliomas. For example, alpha-amino-3-hydroxy-5-methyl-4-isoxazole propionic acid (AMAP)-induced currents promote postsynaptic glioma cell proliferation, which in turn triggers electrical activity in presynaptic neurons through NGSs, and this abnormal electrical activity often manifests as seizures in glioma patients (37, 38). Glioma cells and elevated levels of the excitatory neurotransmitter glutamate could disrupt cerebral cortical networks and provoke tumor-associated epileptic conditions (39, 40). Similarly, in this study, we also found that*MLLT11* upregulated DEGs may be related to the pathogenesis of seizures in glioma-related epilepsy (GRE) patients. Moreover, the*MLLT11* downregulated DEGs mainly clustered in ECM-related pathways, suggesting that the progression of tumor is related to the ECM. The role of the ECM in various cancers has also been proven. For example, the ECM-related pathway is activated and involved in cancer initiation and progression in prostate cancer (41). In gastric cancer, the ECM pathway also plays a pivotal role during tumor invasion and metastasis (42). Also, in colorectal cancer, the ECM participates in the process of epithelial–mesenchymal transition (EMT) and finally leads to tumor malignant progression (43).

In addition, KEGG enrichment analysis was performed on metabolism-related pathways, and the downregulated DEGs were mainly related to leukocyte trans-endothelial migration, complement and coagulation cascades, lysosome, cytokine–cytokine receptor interaction, and ECM–receptor interaction. These pathways are closely related to glioma malignancy. For example, the complement system is considered to be an important system for immune monitoring and homeostasis. It plays an important role in the immune system of organisms. If it is not controlled properly, the immune system can also take action against healthy cells (44, 45). Because glioma patients are often accompanied by lower immunity, we have reason to believe that it is closely related to the complement system. Cytokines are recognized to be key factors in the TME, possessing an immunosuppressive function and inflammatory activity and participating in the progression of GBM (46). Moreover, the ECM–receptor interaction pathways play a role in tumor invasion and metastasis, and the interaction between ECM and the glioma microenvironment is an important contributor to the malignant progression of glioma (47).

Macrophages are the most important immune cells in tumor-related inflammation and play an important role in tumor-related inflammation. At present, macrophages can be divided into two types based on their polarization status: M1-subtype and M2-subtype tumor-associated macrophages (TAMs) (48). M1-subtype TAMs participate in Th1 immune response and have an antitumor effect, while M2 macrophages participate in Th2 immune response and promote tumor growth (49, 50). Previous studies have found that there were a large number of TAMs in GBM. There is a correlation between TAM density and glioma grade, indicating that TAMs support tumor development (51, 52). Interestingly, negative correlations were found in this study between*MLLT11* expression and M2 macrophages, as well as M1 macrophages. It is suggested that, during the progression from low-grade to high-grade glioma, the downregulated*MLLT11* may contribute to recruiting macrophages, which probably mediates the polarization of macrophages to M2, so as to promote tumor progression. In conclusion, our results demonstrate that the expression of*MLLT11* is related to immune cell infiltration and may contribute to the poor prognosis of glioma patients. Further scientific research is needed to explore how glioma cells recruit M2 macrophages during the downregulation of*MLLT11*.

In recent years, immune checkpoint inhibitors (ICI) have brought significant survival benefits to patients in many tumor species. Similarly, preclinical studies have shown that ICIs have great prospects in the treatment of GBM. Therefore, in this study, we also investigated the correlation between*MLLT11* expression and several common immune checkpoint members. Results refer that*MLLT11* had a highly negative correlation with *PDCD1*, *PD-L1*, *TIM3(HAVCR2)*, and PD‐L2 (*PDCD1*LG2) in grade 3 glioma analysis. In GBM,*MLLT11* was strongly negatively correlated with *PD-L1*, *TIM3(HAVCR2)*, and PD‐L2(*PDCD1*LG2). Based on the above confirmed relationship between*MLLT11* and immune checkpoint, we conclude that*MLLT11* has great potential in tumor immunotherapy, and targeting*MLLT11* and other immune checkpoint molecules is likely to be a novel approach for glioma treatment.

In summary, based on the above bioinformatic analysis and experimental validation in glioma, we systematically explored the expression pattern of*MLLT11* according to the clinicopathologic features, molecular subclasses, and prognosis of glioma. Meanwhile, we also analyzed the relationship between*MLLT11* and immune cells in the TME. These results initially illuminate a critical role for*MLLT11* in the progression of glioma. Further studies are needed to explore*MLLT11* as a novel biomarker or therapeutic mediator in glioma, and relevant pharmaceutical studies targeting*MLLT11* will have great potential.

## Data availability statement

Publicly available datasets were analyzed in this study. This data can be found here: UCSC Xena (https://xenabrowser.net/), CGGA website (http://www.cgga.org.cn/), GEO datasets(GSE43378, GSE16011, GSE74187, GSE83300), GTEx databases(http://commonfund.nih.gov/GTEx/).

## Ethics statement

This study was reviewed and approved by Ethic Committee of the Xiangya Hospital of Central South University. Written informed consent for participation was not required for this study in accordance with the national legislation and the institutional requirements.

## Author contributions

LC and ZX were responsible for project design, experiment implementation, data analysis and manuscript drafting. SW and XL were responsible for the revision of manuscript and supervision of the project, also contributed to the discussion. HZ participant experimental design and provided some technical guidance. CT, HL and QH were responsible for collecting clinical samples, assisting in the implementation of the experiment and revising the paper. All authors contributed to the article and approved the submitted version.

## Funding

This work was supported by the National Natural Science Foundation of China (Grant No. 81770781, 81472594) and Natural Science Foundation of Hunan Province, China (Grant No. 2019JJ50978).

## Conflict of interest

The authors declare that the research was conducted in the absence of any commercial or financial relationships that could be construed as a potential conflict of interest.

## Publisher’s note

All claims expressed in this article are solely those of the authors and do not necessarily represent those of their affiliated organizations, or those of the publisher, the editors and the reviewers. Any product that may be evaluated in this article, or claim that may be made by its manufacturer, is not guaranteed or endorsed by the publisher.

## Supplementary material

Supplementary Figure 1 Clinical and molecular characteristics of MLLT11 for gliomas in TCGA and CGGA datasets. **(A, B)** The relationship between age and MLLT11 expression for different WHO pathological grades in CGGA_array and CGGA_693 datasets. **(C-E)** The expression of MLLT11 between female and male in different WHO pathological grades and normal brain tissues. **(C)** TCGA, **(D)** CGGA_325, **(E)** CGGA_693. **(F, G)** Expression of MLLT11 between 1p19q co deletion and non co deletion for grade 2 and 3 gliomas in TCGA dataset. **(H–M)** Expression of MLLT11 between IDH mutant and wild type. MLLT11 expression levels between IDH mutant and wild-type at different WHO pathological grades. **(H, I)** CGGA_325, **(J, K)** CGGA_693, **(L, M)** CGGA_array.

Supplementary Figure 2 Functional enrichment analysis between the MLLT11 high- and low‐expression groups for gliomas in CGGA_325 dataset. **(A)** Differential expression genes (DEGs). **(B, C)** KEGG enrichment analysis of the up-regulated gene in the high MLLT11 subgroup and the down-regulated gene in the high MLLT11 subgroup. **(D, E)** GO analysis of the up-regulated gene in the high MLLT11 subgroup and the down-regulated gene in the high MLLT11 subgroup.

Supplementary Figure 3 Functional enrichment analysis between the MLLT11 high- and low‐expression groups for gliomas in CGGA_array dataset. **(A)** Differential expression genes (DEGs). **(B)** KEGG enrichment analysis of the up-regulated gene in the high MLLT11 subgroup in the high MLLT11 subgroup. **(C, D)** GO analysis of the up-regulated gene in the high MLLT11 subgroup and the down-regulated gene in the high MLLT11 subgroup.

Supplementary Figure 4 Distinct metabolism status in patients with MLLT11 low-expression and high-expression in glioma. **(A)** PCA between the low-expression and high-expression groups based on total DEG in CGGA_325 dataset. **(B)** KEGG pathway enrichment analyses of DEGs in MLLT11 low-expression and high-expression groups in CGGA_325 dataset. **(C)** PCA between the low-expression and high-expression groups based on total DEG in CGGA_693 dataset. **(D)** KEGG pathway enrichment analyses of DEGs in MLLT11 low-expression and high-expression groups in CGGA_693 dataset.

Supplementary Figure 5 Correlation analysis of MLLT11 with 22 immune cells in CGGA_array **(A)** and CGGA_325 datasets **(B)**.

Supplementary Figure 6 Correlation between MLLT11 and seven immune checkpoints in CGGA_325 dataset. **(A)** CD80, **(B)** CD274, **(C)** CD276, **(D)** HAVCR2, **(E)** IDO1, **(F)** PDCD1, **(G)** PDCD1LG2

Supplementary Figure 7 Correlation between MLLT11 and seven immune checkpoints in CGGA_array dataset. **(A)** CD80, **(B)** CD274, **(C)** CD276, **(D)** HAVCR2, **(E)** IDO1, **(F)** PDCD1, **(G)** PDCD1LG2
