# Supplementary figures and images for: Identification of the novel prognostic biomarker, MLLT11, reveals its relationship with immune checkpoint markers in glioma
Source: Front Oncol. 2022 Aug 12;12:889351. doi: 10.3389/fonc.2022.889351 (PMC9414891; doi:10.3389/fonc.2022.889351)

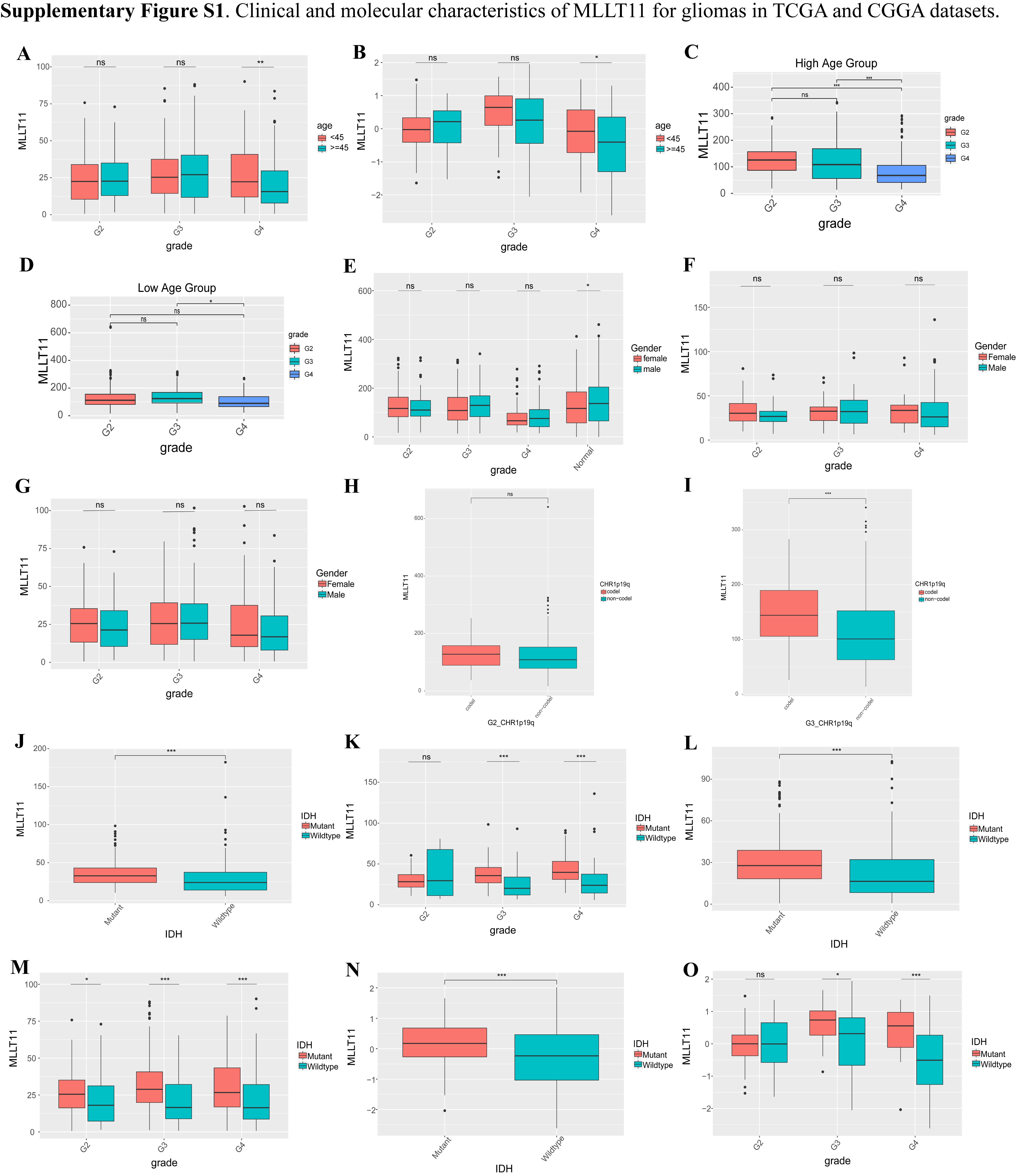

Supplement: Supplementary Figure 1 — Clinical and molecular characteristics of MLLT11 for gliomas in TCGA and CGGA datasets. (A, B) The relationship between age and MLLT11 expression for different WHO pathological grades in CGGA_array and CGGA_693 datasets. (C-E) The expression of MLLT11 between female and male in different WHO pathological grades and normal brain tissues. (C) TCGA, (D) CGGA_325, (E) CGGA_693. (F, G) Expression of MLLT11 between 1p19q co deletion and non co deletion for grade 2 and 3 gliomas in TCGA dataset. (H–M) Expression of MLLT11 between IDH mutant and wild type. MLLT11 expression levels between IDH mutant and wild-type at different WHO pathological grades. (H, I) CGGA_325, (J, K) CGGA_693, (L, M) CGGA_array. [file Image_1.tif]

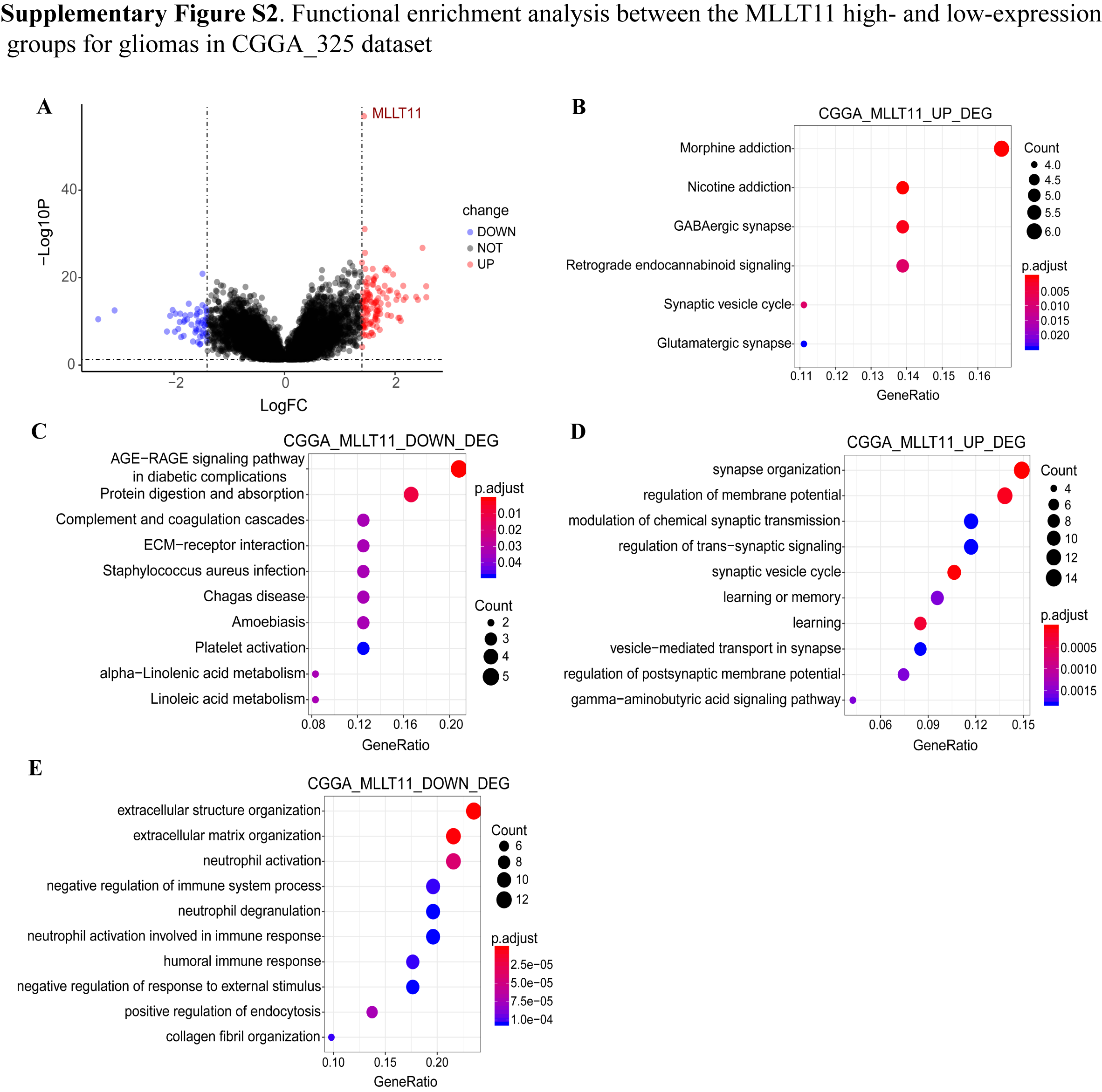

Supplement: Supplementary Figure 2 — Functional enrichment analysis between the MLLT11 high- and low‐expression groups for gliomas in CGGA_325 dataset. (A) Differential expression genes (DEGs). (B, C) KEGG enrichment analysis of the up-regulated gene in the high MLLT11 subgroup and the down-regulated gene in the high MLLT11 subgroup. (D, E) GO analysis of the up-regulated gene in the high MLLT11 subgroup and the down-regulated gene in the high MLLT11 subgroup. [file Image_2.tif]

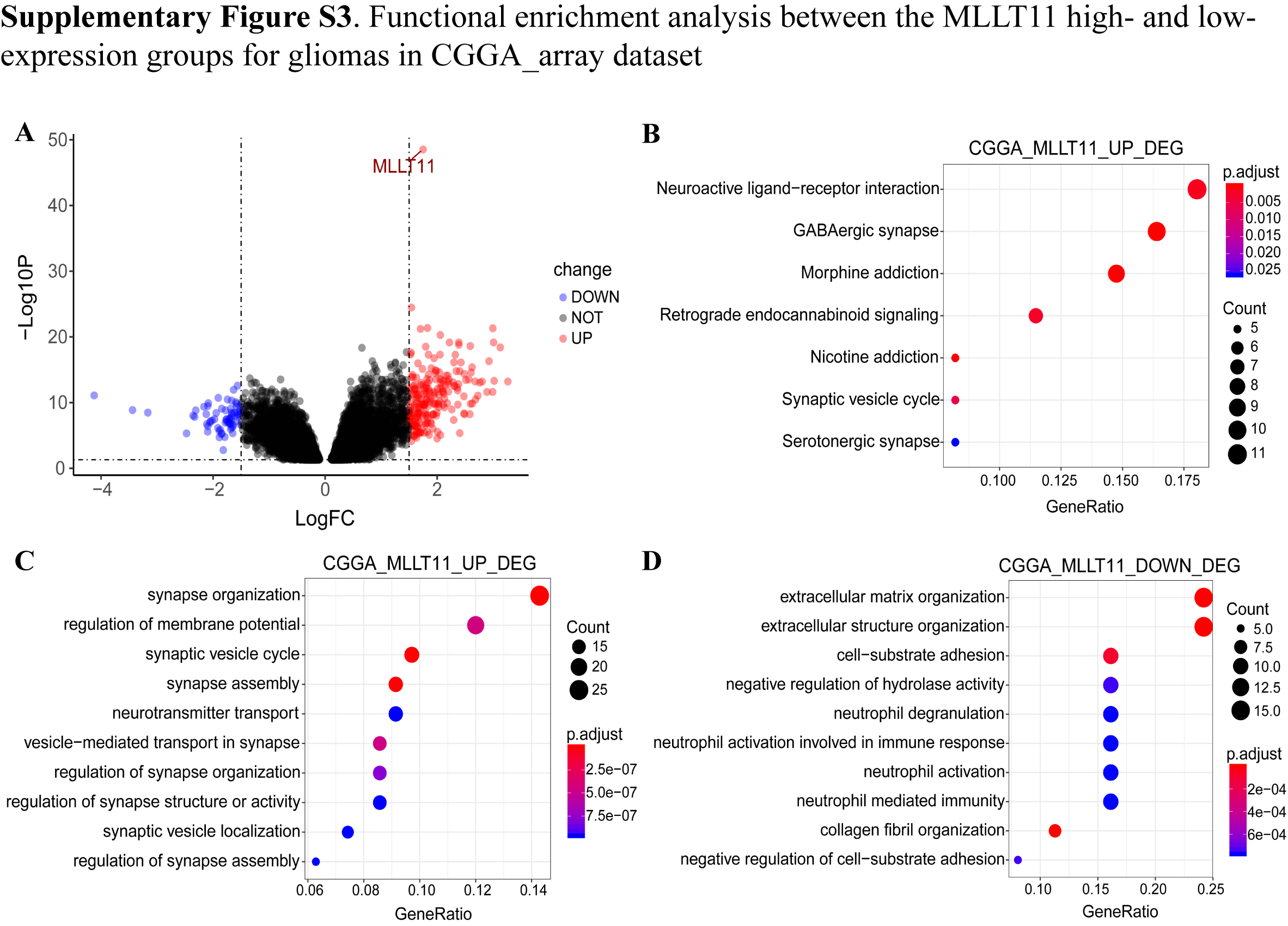

Supplement: Supplementary Figure 3 — Functional enrichment analysis between the MLLT11 high- and low‐expression groups for gliomas in CGGA_array dataset. (A) Differential expression genes (DEGs). (B) KEGG enrichment analysis of the up-regulated gene in the high MLLT11 subgroup in the high MLLT11 subgroup. (C, D) GO analysis of the up-regulated gene in the high MLLT11 subgroup and the down-regulated gene in the high MLLT11 subgroup. [file Image_3.tif]

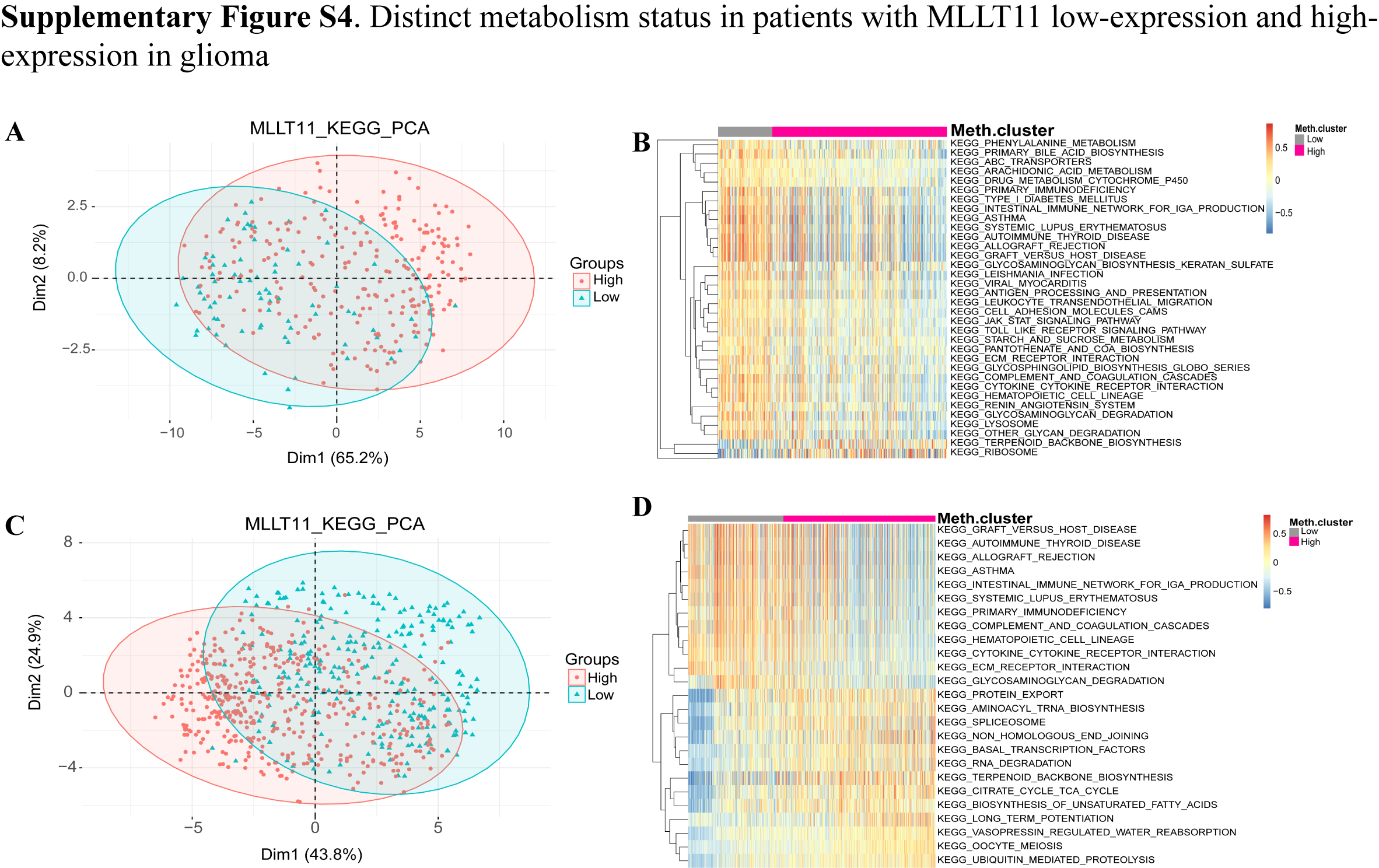

Supplement: Supplementary Figure 4 — Distinct metabolism status in patients with MLLT11 low-expression and high-expression in glioma. (A) PCA between the low-expression and high-expression groups based on total DEG in CGGA_325 dataset. (B) KEGG pathway enrichment analyses of DEGs in MLLT11 low-expression and high-expression groups in CGGA_325 dataset. (C) PCA between the low-expression and high-expression groups based on total DEG in CGGA_693 dataset. (D) KEGG pathway enrichment analyses of DEGs in MLLT11 low-expression and high-expression groups in CGGA_693 dataset. [file Image_4.tif]

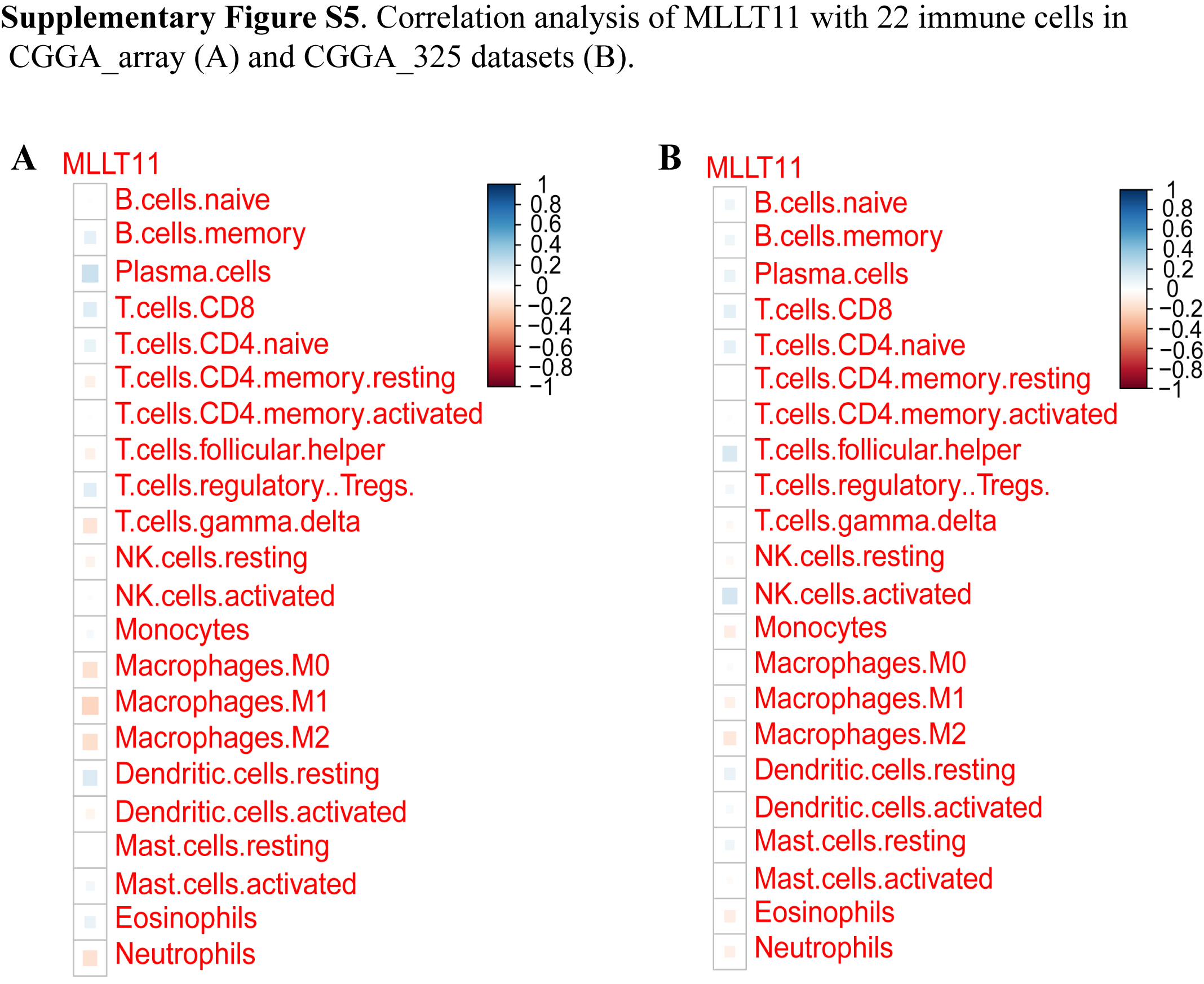

Supplement: Supplementary Figure 5 — Correlation analysis of MLLT11 with 22 immune cells in CGGA_array (A) and CGGA_325 datasets (B). [file Image_5.tif]

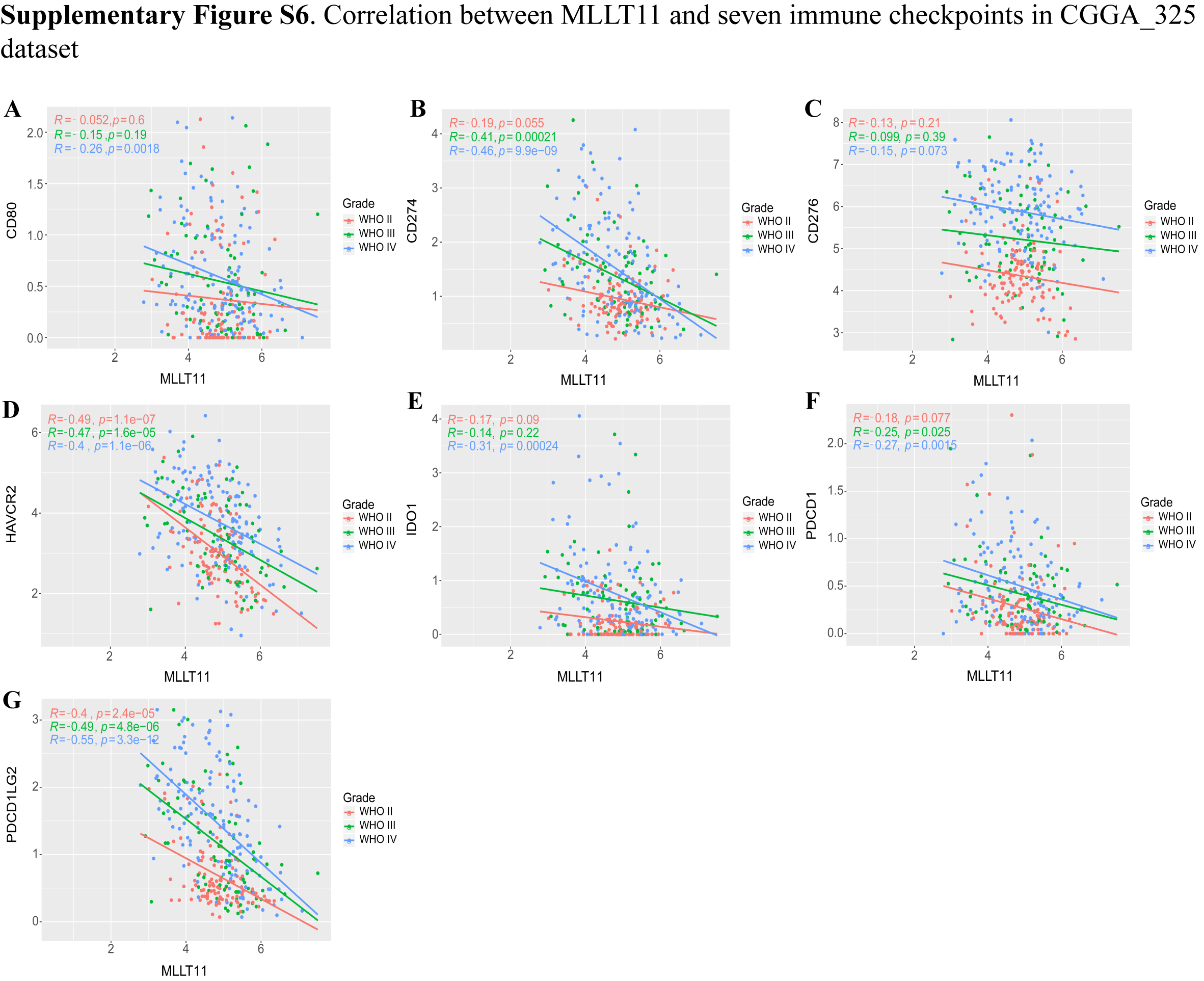

Supplement: Supplementary Figure 6 — Correlation between MLLT11 and seven immune checkpoints in CGGA_325 dataset. (A) CD80, (B) CD274, (C) CD276, (D) HAVCR2, (E) IDO1, (F) PDCD1, (G) PDCD1LG2 [file Image_6.tif]

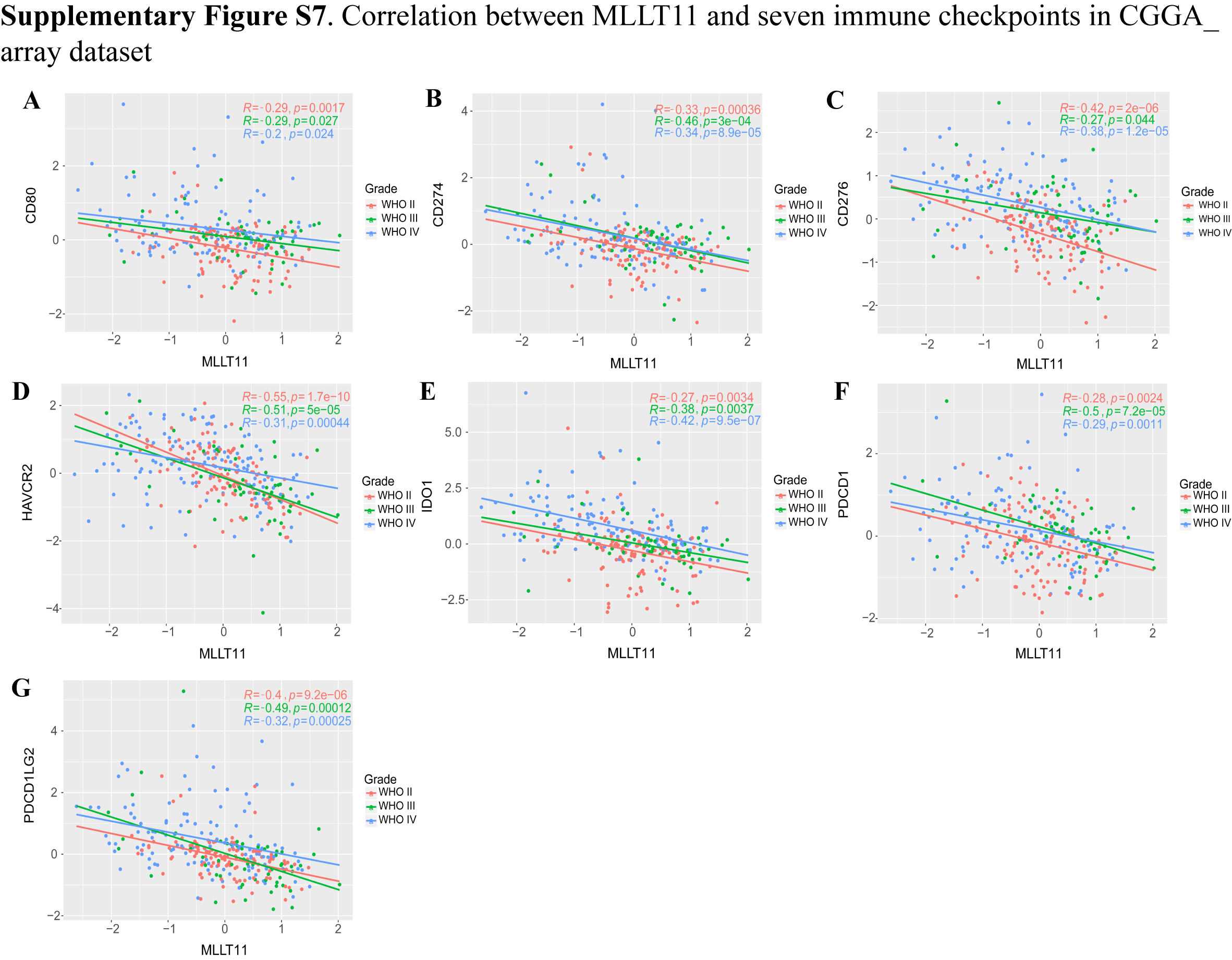

Supplement: Supplementary Figure 7 — Correlation between MLLT11 and seven immune checkpoints in CGGA_array dataset. (A) CD80, (B) CD274, (C) CD276, (D) HAVCR2, (E) IDO1, (F) PDCD1, (G) PDCD1LG2 [file Image_7.tif]
